# Modern Aspects of Pain in Medicine. II

**Published:** 1893-11-11

**Authors:** 


### Modern Aspects of Pain in Medicine.—II.

It will be remembered that we have shown that the spinal cord may be divided into a number of segments, each of which is, on the one hand, connected with a certain viscus, and, on the other, with a certain area of skin which becomes tender when that viscus is diseased. Some viscera are connected with more than one segment of the cord. This fact, which had been carefully observed by Mackenzie, has been fully investigated by Head for nearly every viscus, and the following are some of the results at which he has arrived.

In diseases of the heart, and more especially in aortic disease, the pain is referred along the first, second, third, and fourth dorsal areas. The first dorsal area extends along the inner side of the upper extremity from just above the elbow to the base of the little finger; on the front of the chest it lies below the clavicle for about the width of a rib; the part of it on the back is small, of irregular shape, and lies just above the upper border of the scapula. The second dorsal area on the back cuts the first into two parts; from the axilla it extends down the back of the arm; on the front of the chest it lies below the first dorsal area, but does not extend so far forward. The third dorsal area behind lies below the first and second; it extends down the inner side of the forearm; on the front of the chest it lies below the first and in front of the second. The fourth dorsal area behind lies below the third; it extends well into the axilla, and runs forward as a narrow band beneath the second and third as far as the sternum. Thus the whole area of cutaneous tenderness for diseases of the heart is on the front of the chest, bounded above by the lower border of the clavicle, below by a horizontal line drawn a little above the nipple; the upper limit passes into the axilla round to the back, where it terminates at the seventh cervical spine; the lower limit passes round with one curve up into the axilla, and another down to the angle of the scapula to the fourth dorsal spine. This cardiac area is continued down the inner side of the upper extremity as far as the base of the little finger. On the front of the chest the first dorsal area has a tender spot over about the middle of the second rib; the second has one over the outer border of the sternum at the second rib; the third has one below and to the outer side of that of the first; and the fourth has one just above the nipple. Behind, the tender spot for the first area is just outside the first dorsal spine; that for the second is at the upper border of the scapula, that for the third on its spine, and that for the fourth at the junction of its spine with the vertebral border. We have given this cardiac area at some length, for as heart disease is so common it will probably be one of the most frequently used.

In diseases of the œsophagus the pain and tenderness correspond to the distribution of the fifth dorsal area; that is, one which extends nearly horizontally round the body below the fourth just described. Its lower border passes through the nipple and terminates behind at the fifth dorsal spine. Its tender spot in front is just internal to the nipple, and the one behind lies on the middle of the axillary border of the scapula.

Owing to the fact that many gastric disorders do not kill, there are difficulties in the establishment of the gastric cutaneous areas; but those affected with tenderness in gastric troubles appear to be the sixth, seventh, eight, and ninth dorsal. This series is bounded above by the lower border of the fifth area just mentioned, and below by a nearly horizontal line, extending from the twelfth dorsal spine to the umbilicus. In front, the tender spot for the sixth area is just below the nipple, that for the seventh just below the ensiform cartilage, that for the eighth just below the sixth, and that for the ninth in the nipple line, about the top of the ninth rib. Behind, the tender spot for the sixth area is at the angle of the scapula; and those for the seventh, eighth, and ninth are below it in their respective order.

The tender area associated with gall stones is the eighth, with, of course, its tender spots.

In cases of renal calculus the tender area is the tenth dorsal, which is bounded above by the ninth, and below by a line drawn from the third spine to a point midway between the umbilicus and pubes; this line is not quite horizontal, for it has a slight downward prolongation in its middle. Head gives in full the tender areas corresponding to diseases of the pelvic organs both male and female, but space prevents us from giving them here. Those that we have described are quite sufficient to show the immense importance of this research to clinical medicine. With regard to the serous cavities he comes to the conclusion that affections of them do not cause referred pain, or cutaneous tenderness, but produce local pain which follows the lines of peripheral nerves, and is associated with deep tenderness over the affected point only. It will doubtless strike many of our readers that this statement is open to question, for it is no uncommon thing to meet with referred pain in pleurisy.

Having thus by clinical medicine worked out the sensory supply of the viscera from different segments of the cord, Head next points out that his results agree very closely with those to which Edgworth arrived by anatomical methods, and that also they bear a very curious resemblance to Gaskell's scheme of the motor and inhibitory supply of the viscera. Putting all results together, it appears probable that the sensory fibres of the viscera enter the central nervous system in three great groups. The highest of these is in the head and neck, the middle group lies between the first dorsal and the first lumbar segments of the spinal cord; the lowest group extends from the fifth lumbar to the fourth sacral. Thus there are two gaps in the cord, which are not in connection with sensory fibres from the viscera. The higher of these consists of the fifth, sixth, seventh, and eighth cervical segments, whilst the lower corresponds to the second, third, and fourth lumbar segments.

Now it will, no doubt, have struck the reader that often pain is very wide-spread in diseases of single organs; for instance, disease of the ovary may cause pain far beyond the cutaneous area of its spinal segment, and often disease of one organ, as the gall-bladder, will cause pain and cutaneous tenderness on both sides of the body. To take the latter phenomenon first, it is quite likely to be due to the fact that to a certain extent each viscus is supplied by nerves from both sides of a spinal segment; indeed, when we remember how probably each movement is represented in both cerebral hemispheres the suprise is that pain and tenderness due to disease of one organ is not more often bilateral.

The principle to guide us in the second difficulty, namely, that of the cause of the wide-spread extent of pain due to disease of a single organ, is that nervous impulses very much tend to spread through the central nervous system, just as an electric current will diffuse itself unless rigidly insulated. We have an instance in the fact that if a patient be suffering from Jaksonian epilepsy due to a small local lesion, the convulsions will be first noticed and most manifest in the muscles corresponding to the area implicated in the lesion, but later on in the fit other muscles are convulsed, or as we express it, the cortical discharge has spread. Now if the general health of the patient is low, the resistance of the central nervous system is lowered, and consequently impressions arriving at the spinal segments from the viscera, spread to other segments than that directly connected, in the healthy subject, with the diseased viscus, and consequently the pain and tenderness are experienced not only in the area of skin corresponding to the segment connected with the diseased organ, but in those connected with the segments to which, because of the poor general health, the sensory symptoms have diffused themselves. Some segments and their corresponding cutaneous areas appear to have a lower resisting power than others; for instance, in women the tenth dorsal, the sixth dorsal, and the gastric areas are of a very low resisting power, and consequently, when from anæmia or menstruation, or any other cause, the general resistance of the spinal segments and their cutaneous areas is lowered, it is very common, even when the primary disease of the organ affected is slight, to get secondary pains referred to these areas, hence the frequency of secondary pain in dysmenorrhœa and of vague general pains in anæmic women. If this view is correct, we must clearly treat not only the cause of the pain, but also the anæmia. Another common cause for a lowering of resistance of spinal segments and cutaneous areas is a rise of temperature, and hence the frequency of general pains and aches in febrile diseases.

With regard to the question why pain and tenderness are referred in visceral disease, Head points out that it is probably a psychial process, for when owing to disease of a viscus the higher cerebral centres on receiving apparent impulses from this viscus are so unaccustomed to receiving painful impressions from it, and so accustomed to refer all painful impressions from that segment of the cord to the skin, that they do so now, and hence the pain due to disease of the viscus is referred to the skin. His explanation of the tenderness of the skin is very similar.

